# Local Acidification Limits the Current Production and Biofilm Formation of *Shewanella oneidensis* MR-1 With Electrospun Anodes

**DOI:** 10.3389/fmicb.2021.660474

**Published:** 2021-06-14

**Authors:** Johannes Erben, Zachary A. Pinder, Malte S. Lüdtke, Sven Kerzenmacher

**Affiliations:** ^1^Center for Environmental Research and Sustainable Technology (UFT), University of Bremen, Bremen, Germany; ^2^Electrochaea GmbH, Planegg, Germany

**Keywords:** local acidification, *Shewanella oneidensis* MR-1, biofilm, mass transport, electrospinning

## Abstract

The anodic current production of *Shewanella oneidensis* MR-1 is typically lower compared to other electroactive bacteria. The main reason for the low current densities is the poor biofilm growth on most anode materials. We demonstrate that the high current production of *Shewanella oneidensis* MR-1 with electrospun anodes exhibits a similar threshold current density as dense *Geobacter* spp biofilms. The threshold current density is a result of local acidification in the biofilm. Increasing buffer concentration from 10 to 40 mM results in a 1.8-fold increase of the current density [(590 ± 25) μA cm^−2^] while biofilm growth stimulation by riboflavin has little effect on the current production. The current production of a reference material below the threshold did not respond to the increased buffer concentration but could be enhanced by supplemented riboflavin that stimulated the biofilm growth. Our results suggest that the current production with *S. oneidensis* is limited (1) by the biofilm growth on the anode that can be enhanced by the choice of the electrode material, and (2) by the proton transport through the biofilm and the associated local acidification.

## 1. Introduction

As the juggernaut of climate change is bearing down on mankind, potentially carbon neutral technologies, such as bioelectrochemical systems (BES) have become paramount for rescuing the planet. BES employ biological components that act as catalyst for the conversion of organic or inorganic substrates. *Shewanella oneidensis* MR-1 (MR-1) and derived genetically engineered strains can serve as catalyst for various BES applications, e.g., CO_2_ fixation (Le et al., 2018; La Cava et al., 2020), electrode assisted fermentation to broaden the product spectrum of conventional fermentation (Bursac et al., 2017; Förster et al., 2017), and power generation (Biffinger et al., 2008). In these processes, high current densities are required for compact and cost-efficient production units. Different strategies have been employed in the past to increase the current densities with MR-1 through genetic modifications: enhancement of the biofilm formation (Liu et al., 2015; Silva et al., 2020), optimization of the metabolic flux (Li et al., 2018), and improvement of the extracellular electron transfer (Delgado et al., 2019).

Typically, achievable current densities with MR-1 are far lower than with other electroactive microbes, such as *Geobacter sulfurreducens* (Kipf et al., 2014) but can be improved by the choice of the anode material (Kipf et al., 2013; Patil et al., 2013; Pötschke et al., 2019). The current densities reported in the above publications are substantially lower than the current density achieved with an electrospun carbon nanofiber anode material (ES300), recently developed by our group, and are presumably limited by the anode materials. The superior current production of ES300 [(255 ± 71) μA cm^−2^] is directly linked to the biofilm growth on the anode (Erben et al., 2021b). Analysis of the biofilm revealed that the biofilm fills only about 8 % of the material's pore space and that the available internal surface area is not completely covered with cells. Hence, the question arises what limits the biofilm growth and current production of MR-1. In this context, it is important to note that the above mentioned material studies use an electrode configuration that exposes one side to the growth medium and prevents media flow through the electrode. The current densities achieved in other studies that employ freely mounted electrodes, such as Wu et al. (2020), use flow-cells (Arinda et al., 2019), or undefined anode potentials are not comparable.

Redox mediators, such as flavins and quinones enhance the current production of MR-1. Flavins have been reported to act as electron shuttles enabling mediated electron transfer (Brutinel and Gralnick, 2012) and, more recently, identified as bound co-factor for the terminal reductases MtrC and OmcA enabling faster direct electron transfer (Okamoto et al., 2015). The addition of riboflavin (free or covalently bound to carrier beads) increase biofilm formation and current production (Arinda et al., 2019). Wu et al. (2020) investigated the effect of high concentrations (50 μM) of a variety of mediators. High concentrations of mediators enable higher contributions of mediated electron transfer to the total current production in addition to increased biofilm formation. Lower mediator concentrations (~1 μM) are secreted by MR-1 under micro-aerobic conditions (Erben et al., 2021b). Micro-aerobic conditions enhance the current production at the cost of reduced product yield (Teravest et al., 2014) through enhanced the biofilm formation (Erben et al., 2021b).

Diffusive proton transport out of the biofilm has been shown to ultimately limit the current density of *Geobacter* spp dominated dense biofilms due to local acidification (Torres et al., 2008). Limitation of the biofilm's current production by local acidification occurs at high current densities that cause steep pH gradients within the biofilm. The phenomenon has been measured using pH micro-electrodes in *Geobacter sulfurreducens* biofilms at current densities of 163 μA cm^-2^ and 287 μA cm^-2^ (Babauta et al., 2012). MR-1 biofilms producing ~5 μA cm^-2^ did not show a pH-drop (Babauta et al., 2011). Local acidification is limited to the interior of the anodic biofilm and does not affect the bulk pH. Increasing buffer concentrations, that do not alter the bulk pH, enhance proton transport and enable higher current densities. This way, the effect of local acidification can be assessed indirectly by the current production response to changing buffer concentrations. In the dense biofilms *Geobacter* spp, grown in unbuffered growth media, current densities above the threshold of 194 μA cm^-2^ cause local acidification (Torres et al., 2008).

Mass transport in non-flow-through electrodes is governed by diffusion. In phosphate buffered growth media, the total diffusive proton flux *j* is carried by H_2_${\text{PO}}_{4}^{-}$. The contribution of H^+^ can be neglected in the pH range 5–8 at the buffer concentrations used in this study. At steady state the proton flux can thus be described by Fick's first law

(1) $$\begin{array}{r} j_{{\text{H}}_{2}{\text{PO}}_{4}^{-}}=-D^{\text{rel}}D\nabla c_{{\text{H}}_{2}{\text{PO}}_{4}^{-}}. \end{array}$$

Here, $\nabla c_{{\text{H}}_{2}{\text{PO}}_{4}^{-}}$ is the concentration gradient of the buffer molecules H_2_${\text{PO}}_{4}^{-}$. The concentration gradient $\nabla c_{{\text{H}}_{2}{\text{PO}}_{4}^{-}}$ responds to increasing buffer concentrations resulting in increased proton flux at higher concentrations. The relative diffusion coefficient *D*^rel^ accounts for the reduced diffusion coefficients in the biofilm/electrode composite compared to the bulk diffusion coefficient *D* of H_2_${\text{PO}}_{4}^{-}$ · *D*^rel^ is a dimensionless factor that can assume values between zero (no transport by diffusion) and one (no reduction of the diffusive transport). In the biofilm/electrode composite, the relative diffusion coefficient is reduced by the biofilm in the pore space and the electrode itself. The relative diffusion coefficient of MR-1 biofilms is about 0.8 (average value of profiles measured by nuclear magnetic resonance imaging Renslow et al., 2010). This value is further reduced by the electrode. The reduction by the electrospun electrode can be calculated from the electrode's porosity (0.95 %, Erben et al., 2021b) using Equations (5) and (8) in Inoue et al. (2016) to a value of about 0.9. The effective value of *D*^rel^ in the biofilm/electrode composite of about 0.7 is given by multiplication of the biofilm's and electrode's relative diffusion coefficients. This estimated value of 0.7 for the MR-1/electrode composite is considerably higher than the value of 0.14 for dense *Geobacter* spp. biofilms obtained by simulation (Marcus et al., 2011) and the average values of ~0.4 to ~0.5 for *Geobacter sulfurreducens* biofilms measured by magnetic resonance imaging (Renslow et al., 2010). As a consequence, one could assume that the threshold current of the MR-1/electrode composite is considerably higher than the ~194 μA cm^-2^ of *Geobacter* spp. biofilms. However, the MR-1 biofilm structure differs greatly from the dense, 170 μm–370 μm thick (Renslow et al., 2010; Marcus et al., 2011), *Geobacter* spp. biofilms: the biofilm is less dense, fills only about 8 % of the pore space, and extends through the full 500 μm thick electrode (Erben et al., 2021b). This leads to reduced gradients $\nabla c_{{\text{H}}_{2}{\text{PO}}_{4}^{-}}$ that result in a lower diffusive flux and current production. It is therefore unclear whether the effect of the higher relative diffusion coefficient or smaller gradients predominates the threshold current of the MR-1/electrode composite set by local acidification.

In the present work, we investigate the effect of local acidification on the current production of MR-1/electrode composites using varying buffer concentrations. A custom electrospun material (ES300) with a current production above and a commercial material (C-Tex 13) with a current production below the acidification threshold of *Geobacter* spp. biofilms serve as anode materials. Riboflavin is used to stimulate biofilm growth and the current production. To exclude limitations imposed by nutrient depletion and MR-1 cell abundance (cell density), complementary experiments were performed.

## 2. Materials and Methods

### 2.1. Bioelectrochemical Characterization

The current production was recorded in half-cell configuration at -41 mV vs. a saturated calomel electrode (KE 11, Sensortechnik Meinsberg, Germany) at 30 °C using potentiostats (PGU-MOD 500mA, IPS Elektroniklabor GmbH & Co KG, Germany). A platinum mesh served as counter electrode. The bioelectrochemical reactor holds 1 L growth medium and six places for working electrodes (Erben et al., 2021b). The anodes are mounted in holders that expose 2.25 cm^2^ to the growth medium. The growth medium was stirred with a magnetic stir bar at 300 rpm. The reactor headspace was continuously purged with N_2_ at ~1 L min^-1^. The reactor and the growth medium were sterilized at 121°C for 20 min. Polarization of the anodes and anaerobization overnight prior to inoculation ensures negligible non-faradaic currents and reductive currents from residual oxygen. An illustration of the reactor can be found in Supplementary Figure 1. The biolectrochemical reactor configuration features a conductivity dependent uncompensated resistance of *R*_U_ = *ρ*_U_/*σ*. The parameter *ρ*_U_ of 55 m^-1^ reflects our reactor design. For a given growth medium conductivity *σ*, the potential deviation at the anode due to the ohmic drop in the growth medium is given by Δ*U* = *R*_U_ · *A* · *i* (electrode area *A* and the current density *i*). The highest potential deviation in this work is 34 mV and occurs at a current density of *i*= 590 μA cm^-2^ with a medium conductivity of 21.2 mS cm^-1^ and is therefore negligible.

### 2.2. Anode Materials

Tailored electrospun carbon fiber mats with an average fiber diameter of 286 nm (ES300) and a commercial knitted activated carbon fabric (C-Tex13 , MAST Carbon, Basingstoke, UK) served as anode materials. ES300 was previously identified as material with the highest current production of MR-1 with the reference medium described in Section 2.3. C-Tex13 exhibits a lower current production and serves as reference material (Erben et al., 2021b). The fabrication process of ES300 was described in detail by Erben et al. (2021a).

### 2.3. Growth Media

A phosphate buffered saline (10 mM PBS) growth medium with 50 mM DL-lactate as electron source, previously used in several studies (Golitsch et al., 2013; Kipf et al., 2013; Dolch et al., 2014), served as reference growth medium (RM) in this study. The following media were derived from RM:

1. Medium with reduced lactate concentration (25 mM)
2. Two media supplemented with 500 and 1,000 nM riboflavin. Riboflavin was supplemented from a stock (100xRF) prior to the bioelectrochemical characterization through a sterile filter without autoclavation.
3. Improved medium (IM) with 40 mM PBS and 1,000 nM riboflavin. The NaCl concentration was reduced to match the conductivity of RM (20.2 mS cm^-1^).

The lactate concentration was increased by spiking 50 % sodium DL-lactate solution to the medium with reduced lactate concentration. The buffer capacity was increased by spiking 25xPBS medium containing 250 mM PBS to RM. The components of all media used in this study are listed in Supplementary Table 1. The chemicals were obtained from Sigma Aldrich (Taufkirchen, Germany), and Carl Roth (Karlsruhe, Germany).

### 2.4. Cell Cultivation

*Shewanella oneidensis* MR-1 (MR-1) cells from a cryo-stock were spread out on an LB(lysogeny broth)-agar plate. A single colony was picked for aerobic pre-cultivation in LB medium overnight. One hundred microliters of the pre-culture were transferred to anaerobic medium (AM) with fumarate as electron acceptor and cultivated for 24 h. The cells were harvested by centrifugation and washing three times in washing buffer (WB, see Supplementary Table 1) and finally redispersed in RM. The optical density of the inocula ranged between 10 and 20. The volume of the inoculum was adjusted for the targeted initial optical density of 0.05 in the reactor. All cultivation steps were performed at 30°C.

### 2.5. Statistical Analysis

The spike experiments are evaluated before the first spike on Day 6, and 2 days after each spike at Day 8 and 10. The effect size of the parameters is quantified as log_2_ fold change:

(2) $$\begin{array}{r} \log_{2}\left(\text{foldchange}\right)=\log_{2}\left(\frac{i_{k}}{i_{\text{Day6}}}\right);k=\text{Day8, Day10.} \end{array}$$

The effect size of riboflavin addition is measured by the log_2_ fold change of the maximum current density *i*_Max_ relative to the control without riboflavin addition:

(3) $$\begin{array}{r} \log_{2}\left(\text{foldchange}\right)=\log_{2}\left(\frac{i_{\text{Max,}l}}{i_{\text{Max,0 nM}}}\right);l=\text{500 nM, 1,000 nM.} \end{array}$$

The significance levels of the effect size were calculated with a two-tailed Welch corrected *t*-test against the respective control. The *p*-values for the spike experiments were calculated based on the fold changes and the *p*-values for the effect of riboflavin based on the maximum current density.

### 2.6. Biomass Quantification

The dry weight equivalent of the biofilms attached to the anodes was quantified by analysis of the protein content of cell lysate. The individual anodes were placed in 1 mL lysis buffer (LyB, see Supplementary Table 3) for at least 24 h. The protein content was then determined with a colorimetric test (Roti-quant universal, Carl Roth, Germany) using a 96-well plate and a plate reader (Tecan Spark, Tecan Austria GmbH, Austria) according to the manufacturers' instructions. The dry weight equivalent was calculated using a MR-1 standard (filter cake from a cell culture with known volume, cell density, and dry weight equivalent). The dry weight equivalent of the planktonic cells *m* was obtained by {*m*}_mg_ = 716 · {*OD*_600_}.

### 2.7. Experimental Design

In order to increase the power of the statistical analysis of the effect size, spike experiments for the parameters inoculation strength, buffer capacity, and lactate concentration were carried out. This allows us to minimize experimental variability by evaluating current production changes of individual anodes. The first spike was performed after 6 days of initial growth that allow the current production to stabilize. The second spike is performed after another 2 days of equilibration time. Spike experiments with riboflavin failed for unknown reason: the current production peaks after riboflavin addition (see Supplementary Figure 2) and no stable current-read out was possible. Thus, the effect size of riboflavin addition on the current production was determined in individual experiments. Special care was taken to reduce the experimental variability by running the experiments in parallel with media from the same batch. The six holding places in the bioelectrochemical reactor were fitted with two triplicates of ES300 and C-Tex13.

## 3. Results

The aim of this study was to explore the limiting processes of the current production with MR-1. We investigated the current response to varying abundance of MR-1 cells (inoculation strength), electron source (lactate concentration), riboflavin, and local acidification (buffer capacity). To optimize the statistical power of the experiments, spike experiments were performed whenever possible. This was not possible for the parameter riboflavin, since no stable values were obtained. See Section 2.7 for the details.

### 3.1. Current Production

#### 3.1.1. Effect of Buffer Capacity

**Figure 1:**
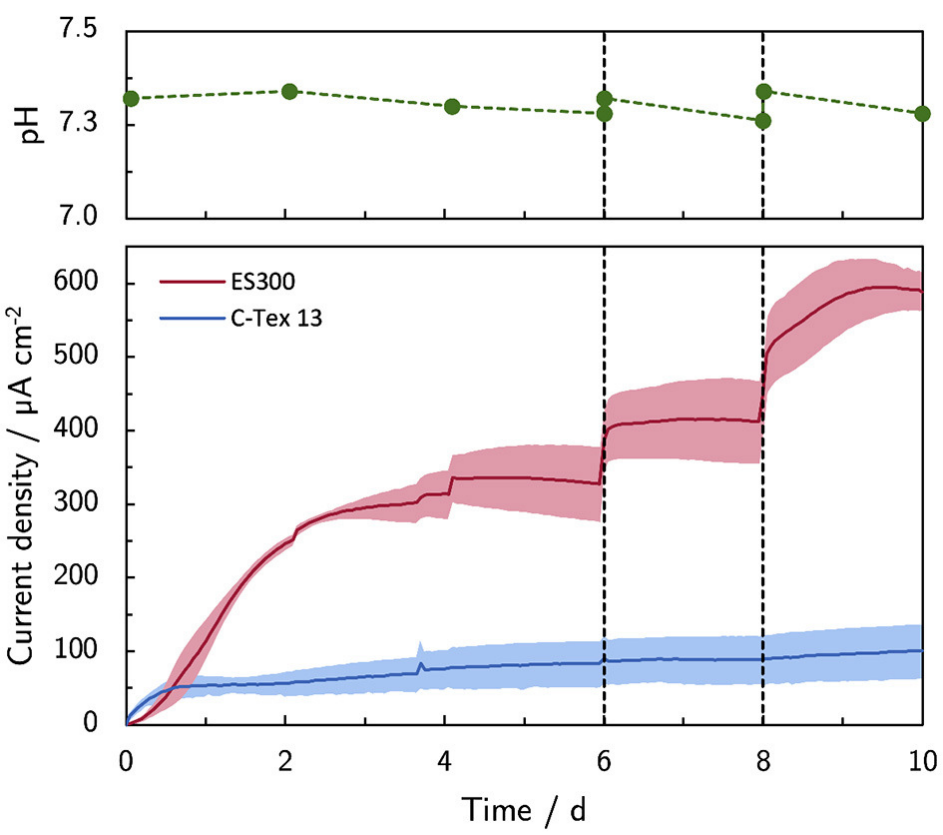
Effect of the buffer capacity on the current production. The initial buffer concentration of 10 mM was increased on day 6 to 20 mM (+10 mM) and on day 8 to 40 mM (+20 mM). Note the stable pH during the experiment. The shaded area corresponds to the sample standard deviation of three anodes. The numerical values of the current densities on day 6, 8, and 10 can be found in Supplementary Table 4.

The addition of PBS on day 6 (+10 mM) and 8 (+20 mM) increases the current production with ES300 from 327 μA cm^-2^ to 590 μA cm^-2^ (Figure 1). The lower current production of C-Tex13 (83 μA cm^-2^–88 μA cm^-2^) does not respond to the increased buffer capacity.

**Figure 2:**
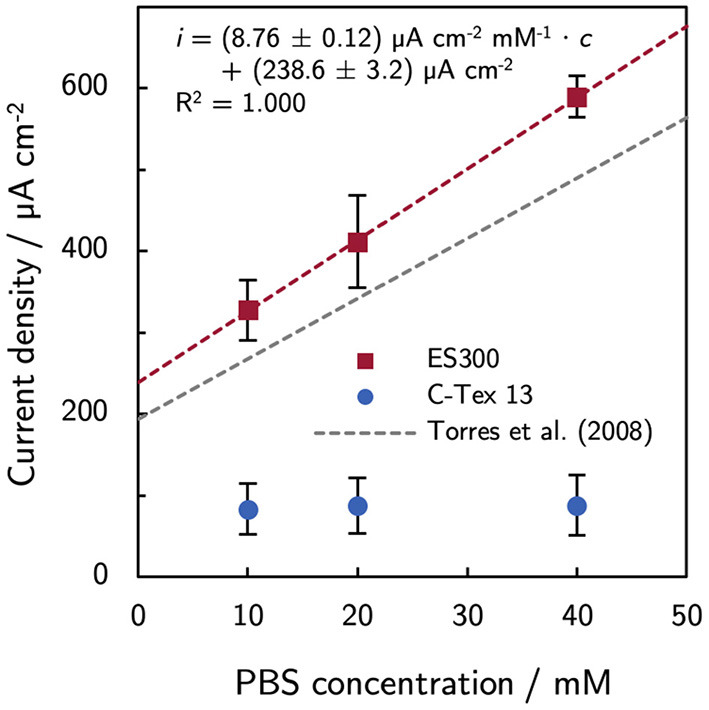
Effect of phosphate buffer concentration on the current production. The current production of ES300 shows a linear response to the buffer concentration. The *y*-axis intercept of 239 μA cm^-2^ corresponds to the threshold current density of the MR-1/ES300 composite. The threshold current density and the slope of the linear response is slightly higher than for dense mixed community biofilms reported by Torres et al. (2008). See Discussion for a details. The numerical values can be found in Supplementary Table 4.

The bulk pH is stable in the range between 7.26 and 7.34 during the experimental time of 10 days. Thus, higher PBS concentrations reduce local acidification caused by the high current density of MR-1 with ES300. The current production with ES300 shows a linear response to the buffer concentration (Figure 2). We define the *y*-axis intercept of 239 μA cm^-2^ as threshold current density. The threshold current density and the slope of 8.75 μA cm^-2^ mM^-1^ relate to the proton flux from the biofilm to the bulk medium.

The conductivity of the growth medium upon buffer addition increases from 20.2 mS cm^-1^ to 21.7 mS cm^-1^. This corresponds to an increase of only 7 % while the current production increases by 80 %. As the medium conductivity is inversely related to the uncompensated resistance in the experimental setup, the increased conductivity cannot explain the observed current increase. The uncompensated resistance in our experimental setup leads to negligible potential deviations of <34 mV (see Section 2.1). The related increase of the ionic strength has no effect on the current production of MR-1 in the range 280 mM–430 mM (Kalathil et al., 2014). Although, the range of the ionic strength in our experiments is not identical (~220 mM to ~305 mM, estimated by the salt contents of the growth media at pH 7.3) we do not expect a substantial impact on our results. This is supported by the current production of (575 ± 63) μA cm^−2^ with improved medium (IM, Supplementary Figure 3, same conductivity as the reference medium RM).

#### 3.1.2. Effect of Riboflavin

**Figure 3:**
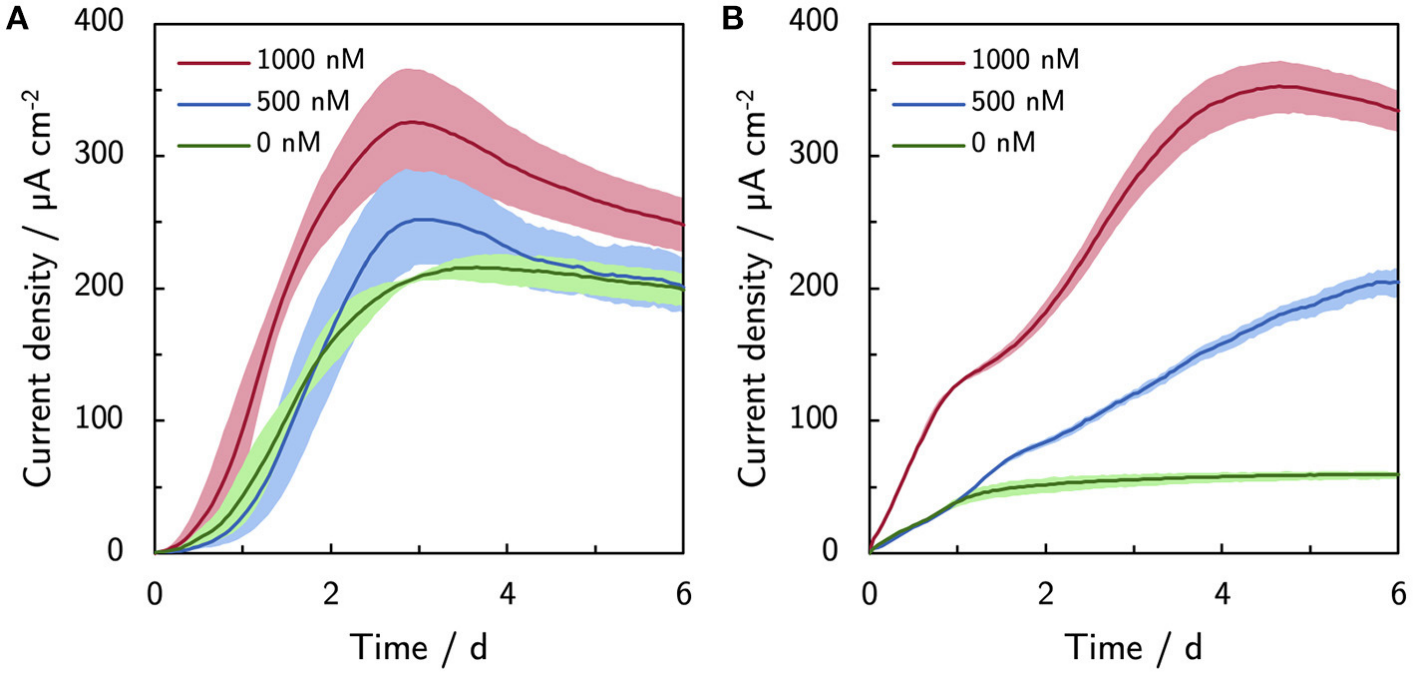
Effect of riboflavin (500 and 1,000 nM) on the current production. **(A)** Current production of ES300 and **(B)** C-Tex 13. The shaded area corresponds to the sample standard deviation of three anodes. The numerical values of the maximum and final current densities can be found in Supplementary Table 4.

Riboflavin was supplemented up to 1,000 nM to mimic the levels of naturally secreted flavins under micro-aerobic conditions Erben et al. (2021b). Supplemented riboflavin increases the current production of ES300 and C-Tex13 (Figure 3). The current production of ES300 in panel A shows an overshoot around day 3 and a subsequent drop to values comparable to the control without supplemented riboflavin. The current production response of C-Tex13 to supplemented riboflavin is considerably different (panel B) in Figure 3. The current production without riboflavin addition stabilizes between day 1 and day 6 at ~60 μA cm^-2^. Five hundred nanomolar riboflavin causes further increase until day 6 and 1,000 nM a current overshoot between day 5 and 6. The current densities with ES300 seem to asymptotically approach a current density close to the current density obtained without supplemented riboflavin. This current density is similar to 203 ± 31 μA cm^−2^ after 14 days of operation in our previous work (Erben et al., 2021b) (value not explicitly stated). This suggests a temporary effect of riboflavin on the current production that is ultimately limited by local acidification.

#### 3.1.3. Effect of Inoculation Strength

**Figure 4:**
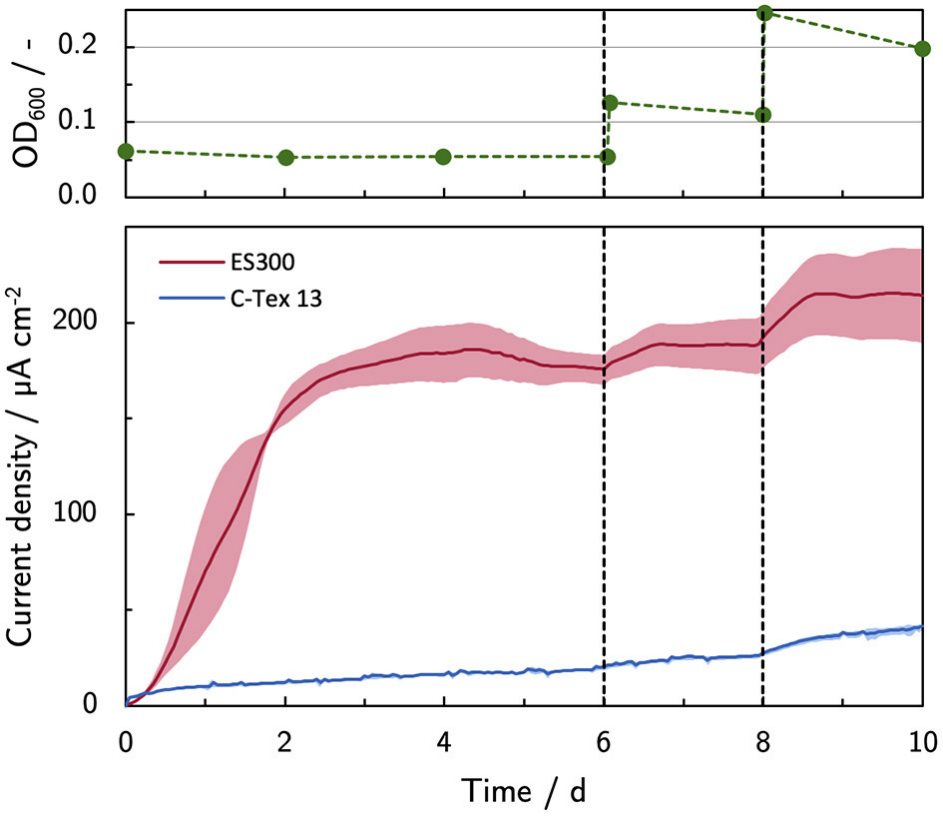
Current production with inoculum spikes on day 6 and 8. The current density is evaluated before each inoculum spike on day 6 and 8, and at the end of the experiment on day 10. The shaded area corresponds to the sample standard deviation of three anodes. The numerical values can be found in Supplementary Table 4.

As reported by Erben et al. (2021b), the current production of MR-1 is directly linked to the cells attached to the anode. The most obvious approach to enhance the biofilm formation is to use a higher inoculation cell density (Figure 4). A cell spike (OD_600_ +0.05) on day 6 stabilizes the current production of ES300 that would otherwise slightly decrease. No effect is observed for the current production of C-Tex 13 . The third inoculation (OD_600_ +0.1) increases the current production of ES300 and C-Tex 13 significantly. The effect is more pronounced for C-Tex 13.

#### 3.1.4. Effect of Lactate Concentration

The lowest lactate concentration of 25 mM is not limiting the current production. Spiking additional 25 mM and 50 mM does not result in a significant increase of the current production compared to the control. A time series of the current production is depicted in Supplementary Figure 4. The total daily lactate consumption under anaerobic conditions (Coulombic efficiency ~97 %, Erben et al., 2021b) with 6 anodes that produce 575 μA cm^-2^ (the maximum with IM) is <2 mmol d^-1^. Thus, during the experimental time of 10 days lactate availability is not limiting the current production with an initial lactate concentration of 50 mM used in the other experiments.

#### 3.1.5. Effect Size

**Figure 5:**
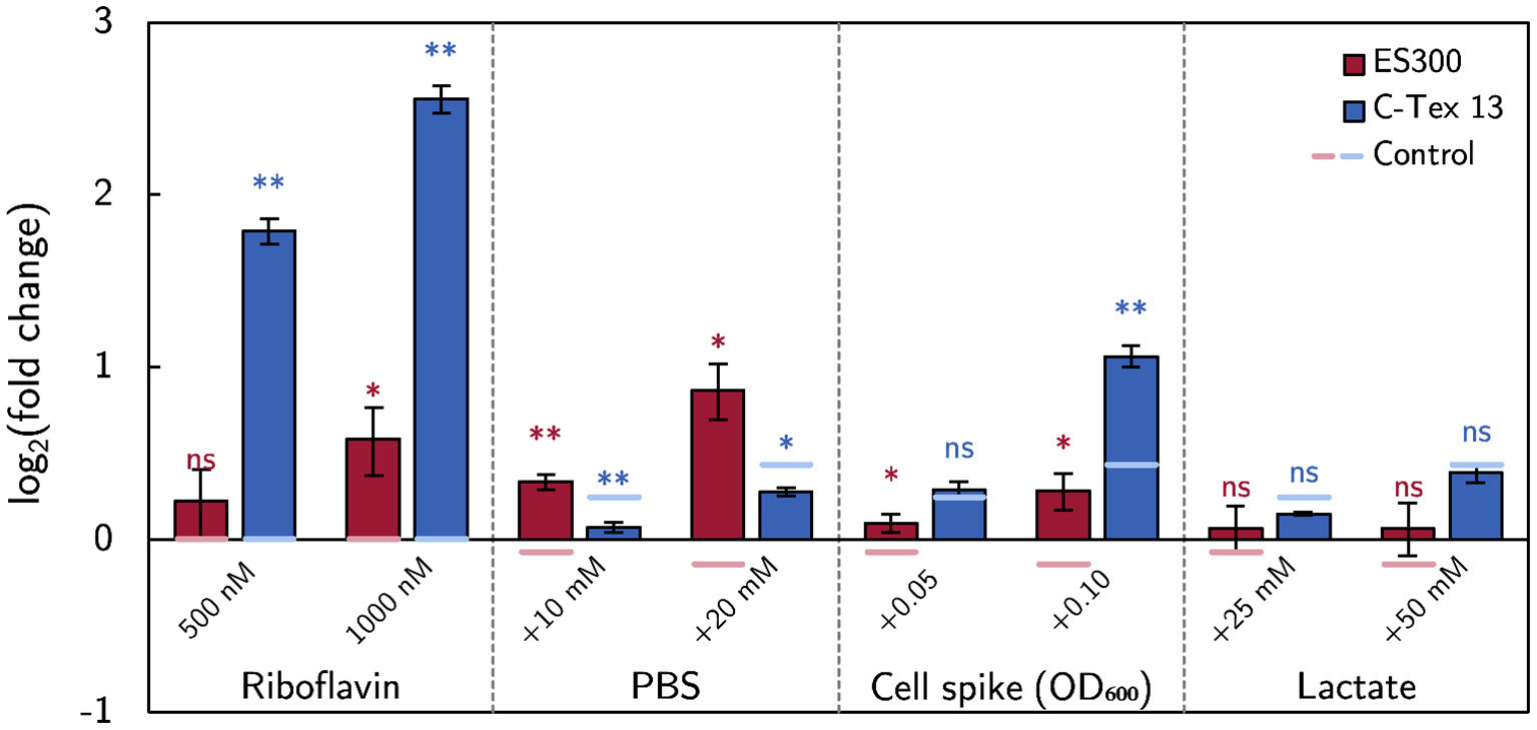
The effect of the medium components on the current production. The effect size is measured as fold change of the current production. The asterisks indicate significance levels: *p* < 0.01 (^**^), *p* < .05 (^*^), *p* ≥ 0.05 (ns). The significance levels of the spike experiments were calculated against the control without spikes (Supplementary Figure 5) and the effect of riboflavin against the current production without riboflavin (Figures 3A,B). The numerical values can be found in Supplementary Table 4.

The effect size of the above described experimental parameters is summarized in Figure 5 as log_2_ (fold change) of the current production compared to the current production on day 6 or with RM in case of riboflavin addition. The strongest effect on the current production of C-Tex 13 has riboflavin, while the buffer capacity has the strongest effect on the current production of ES300. Addition of fresh inoculum has a positive impact on both anode materials. Particularly noteworthy is the fact that riboflavin addition affects mainly the current production of C-Tex 13 that exhibits a low current production without riboflavin addition. ES300 current production has a higher base level and is affected less by riboflavin addition. With ES300 1,000 nM riboflavin supplement enhances the current on day 6 only (1.25 ± 0.13)-fold while C-Tex 13 ' current production is enhanced (5.64 ± 0.26)-fold.

### 3.2. Biofilm Formation

**Figure 6:**
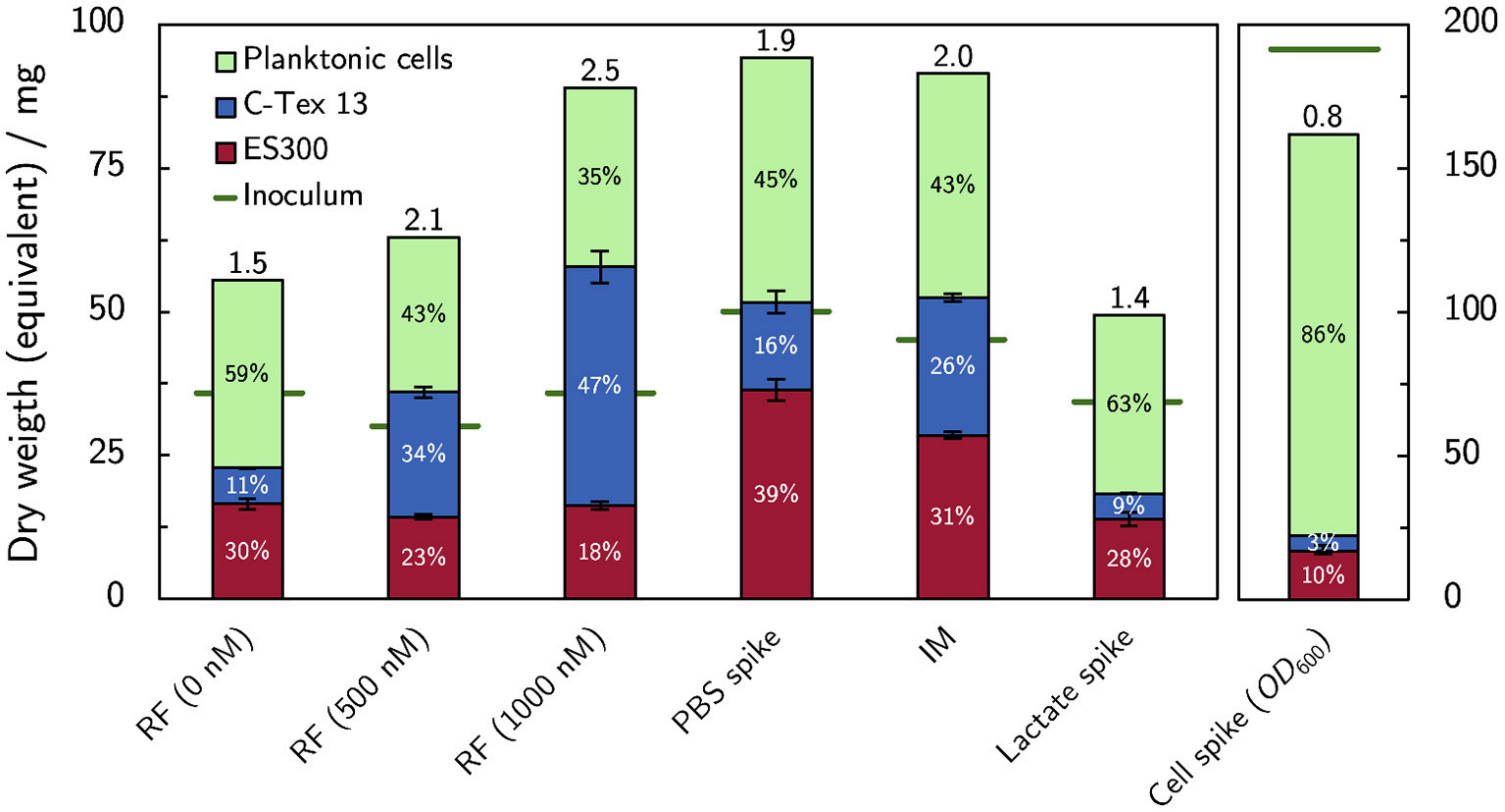
Dry weight equivalents of the biofilm on the anodes and planktonic cells. Addition of flavin enhances biofilm formation on C-Tex 13. PBS addition improves mainly the biofilm formation on ES300. The numbers at the column tops indicate the total growth yield in the reactor (total dry weight equivalent at the end of the experiment divided by the dry weight equivalent of the inoculum). Note the different scale of the inoculation experiment, depicted on the right. The dry weight of the biofilms attached to the anodes (*n* = 3) and the dry weight equivalent of planktonic cells were evaluated at the end of the experiment on day 6 (supplemented RF, IM) or 10 (spike experiments). The numerical values can be found in Supplementary Table 4.

The dry weight equivalent of the biofilms attached to the anodes at the end of each experiment was analyzed together with the dry weight equivalent of the inoculum and the planktonic cells at the end of the experiments. Figure 6 summarizes the cells distribution between anodes as biofilm and planktonic cells. Buffer and riboflavin addition enhance the bacterial growth (expressed as total amount of cells at the end of the experiment divided by inoculum, numbers on top of the columns in Figure 6) in the reactors to 1.9–2.5 compared to the growth of 1.4–1.5 without cell additions. The increase of bacterial growth can be attributed to enhanced biofilm formation: riboflavin enhances biofilm attachment to C-Tex 13, and PBS addition mainly to ES300. The addition of lactate has no impact on the cell growth in the reactor. The biofilm formation with the improved medium IM shows an intermediate behavior: more biofilm is attached to ES300 and C-Tex 13 compared to the reference medium but the total biomass attached to the anodes does not exceed the values of riboflavin and PBS addition. The inoculation with a cumulative inoculation with *OD*_600_ = 0.2 results mainly in excess planktonic cells.

### 3.3. Biofilm Growth and Anode Material Determine the Current Production

**Figure 7:**
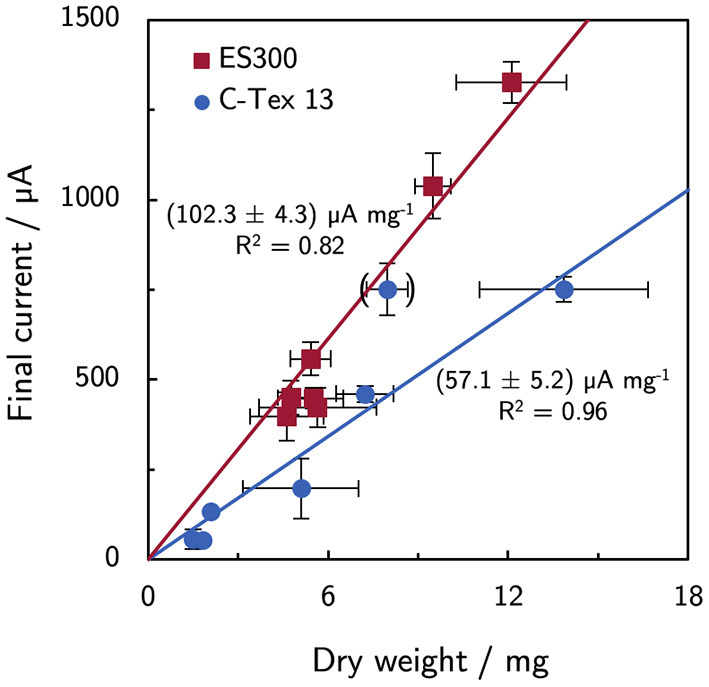
Current and dry weight for all experimental conditions. ES300 enables a higher current production per biomass dry weight as compared to C-Tex 13. The value in brackets was excluded from the linear regression (C-Tex 13 with IM). The reasoning is discussed in the text.

In a previous article by Erben et al. (2021b), a current per dry weight ratio of (65.2 ± 7.0) μA mg^−1^ was determined for electrospun materials with different fiber diameters and additional activation. The value for C-Tex 13 did not deviate significantly. The values were obtained with the reference medium of the present study with a maximum current density of (255 ± 71) μA cm^−2^. In the present study, the higher current densities reveal differences between the two materials (Figure 7): the current to dry weight ratio with ES300 [(102.3 ± 4.3) μA mg^−1^] is significantly higher than the value with C-Tex 13 [(57.1 ± 5.2) μA mg^−1^]. We explain this difference by the less error prone measurement at higher current densities and dry weights in the present work. An exception is the value obtained with IM and C-Tex 13 [(94 ± 17) μA mg^−1^] that does not differ significantly from the values of ES300.

## 4. Discussion

In this study, the current density of MR-1/ES300 could be improved by increasing the buffer capacity of the growth medium. The response to increasing PBS concentration follows the linear relation *i* = (238.6 ± 3.2) μA cm^−2^ + (8.76 ± 0.12) μA cm^−2^ mM^−1^ · *c*_PBS_. The increasing current density can be attributed to enhanced proton transport by the buffer. Torres et al. (2008) observed a similar response of mixed community biofilms (*i* = 194 μA cm^-2^ + 7.4 μA cm^-2^ mM^-1^ · *c*_PBS_). Both, the threshold current density and the slope of the linear response are slightly higher by a factor of about 1.2. The higher values are related to a higher proton flux (Equation 1) and can be a result of a higher relative diffusion coefficient *D*^rel^ and/or a smaller effective thickness of the biofilm in the anode. The effect of increased buffer concentration is limited to anodes with innately high current production as the buffer concentration does not affect the current production of C-Tex 13. The biofilm structure on ES300 was previously studied (Erben et al., 2021b) and shows biofilm penetration to the backside of the electrode. Hence, we tend to attribute the 1.2-fold higher values to a higher relative diffusion coefficient. At pH 7, the buffer reduces the impact of the proton release on the pH mainly through the reaction ${\text{HPO}}_{4}^{2-}+{\text{H}}^{+}↔{\text{H}}_{2}{\text{PO}}_{4}^{-}$. Metabolic activity of MR-1 has been demonstrated at pH5 (Biffinger et al., 2008) and reaches possibly to even lower pH values. Under anaerobic conditions, lactate that is metabolized incompletely to acetate (${\text{Lactate}}^{-}+{\text{H}}_{2}\text{O}\to {\text{Acetate}}^{-}+{\text{CO}}_{2}+4{\text{H}}^{+}+4{\text{e}}^{-}$). Acetate (p*K*_a_ = 4.8) may contribute to the buffer capacitance in the pH range 5–6 within the biofilm. However, acetate accumulation in our reactor is only about 7 mM [estimated based on the Coulombic efficiency of 97 % determined for our experimental setup (Erben et al., 2021b) and the total current production in the reactor]. Therefore, the contribution of acetate to the buffer capacity is presumably negligible. As a side-note: the oxidation and reduction of riboflavin is proton-coupled ($\text{RF}+2{\text{H}}^{+}+2{\text{e}}^{-}↔{\text{H}}_{2}\text{RF}$) and acts therefore as buffer as well. However, the concentrations of riboflavin in this work are at least three orders of magnitude lower than the PBS concentrations. The contribution of riboflavin to the buffer capacity can therefore be neglected.

The addition of riboflavin increased mainly the current production of C-Tex 13. At current densities higher than about 200 μA cm^-2^ an overshoot of the current production is observed. As the increasing current production of C-Tex 13 is directly related to enhanced biofilm formation (see Section 3.2), the reason for the current production overshoot could be biofilm dispersal following a current production at unsustainable levels similar to the reaction of MR-1 biofilms to a decrease in oxygen concentration (Thormann et al., 2005) or cell death. The hypothesis of cell death is supported by our cell spike experiment (Figures 4, 5) in which the current production could be stabilized by cell addition to the reactor. In a study by Wu et al. (2020) a similar behavior with mediator (flavins and quinones) supplemented growth media was reported. In their study, biomass attachment to the anode kept increasing after the current peaks at around day 1 after inoculation. Increasing biomass in the anode pore space lower the relative diffusion coefficient *D*^rel^ and thus the diffusive flux. This finding, as well as the similar asymptotic current density with ES300 and riboflavin supplement of about 200 μA cm^-2^ suggest that flavins enable elevated and stable current densities up to the limit set by local acidification. Interestingly, the combination of 40 mM PBS and 1,000 nM riboflavin did not improve the current production of ES300 and C-Tex 13 compared to the individual components. As the current overshoot was also observed in spike experiments with C-Tex 13 and ES300 (see Supplementary Figure 2) we can exclude the differing material morphologies as cause for the current overshoot. The clarification of the reasons for the current overshoot with supplemented riboflavin would require the in depth study of biofilm dynamics and mass transport that is beyond the scope of this work. In our previous study (Erben et al., 2021b), we observed higher current productions with electrospun anode materials with thicker fibers (ES400 and ES600) and similar current densities with thin fiber materials (ES100 and ES300) under micro-aerobic conditions compared to anaerobic conditions. The micro-aerobic conditions caused flavin secretion of about 1 μM and enhanced the biofilm formation. Considering the findings of the present study, it is likely that the current production with electrospun materials under micro-aerobic conditions is limited by local acidification.

The small effect of multiple inoculations shows that the biofilm formation is self-limiting. Saville et al. (2011) reported that MR-1 biofilm stability requires metabolic activity that is inhibited at low pH (Biffinger et al., 2008). Thus, biofilm attachment could be limited by pH gradients in the biofilm.

Distinct current per dry weight ratios for ES300 [(102.3 ± 4.3) μA mg^−1^] and C-Tex 13 [(57.1 ± 5.2) μA mg^−1^] were found. In a previous work (Erben et al., 2021b), lower current to dry weight ratios were attributed to large macropores of the anode material (GFD 2) that require electron transfer over longer distances and reduce the average metabolical activity. The ratio increased under micro-aerobic conditions that cause elevated flavin secretion. Riboflavin enhances the electrical conductivity of MR-1 biofilms (Pirbadian et al., 2020), reduces the charge transfer resistance (Arinda et al., 2019), and could act as electron shuttle for planktonic cells (Brutinel and Gralnick, 2012). However, these effects would result in increasing current to dry weight ratios upon flavin addition only, which is not observed. Surprisingly, the current to dry weight ratio of C-Tex 13 with IM [(94 ± 17) μA mg^−1^] is similar to the values of ES300 suggesting a synergistic effect of riboflavin and increased buffer capacity. Teal et al. (2006) reported changing metabolic activity at different stages of the biofilm development as response to the local microenvironment. IM might enable a microenvironment on C-Tex 13 that is better suited for current production.

Porous biofilm structure also allow convective transport inside the electrode that enhance the proton transport. In this context, it would be of high interest to quantify the exact pH profile in electrodes populated with MR-1, which is however beyond the scope of the present study. In previous works, local acidification has been quantified directly through pH microelectrode measurements in biofilms attached to non-porous electrodes. Decreasing pH was successfully measured in *Geobacter sulfurreducens* biofilms at current densities from ~160 μA cm^-2^ to ~300 μA cm^-2^ (Babauta et al., 2012). MR-1 biofilms did not reveal pH gradients—presumably due to the low current densities of ~5 μA cm^-2^ (Babauta et al., 2011). Another approach to assess pH-profiles are complex metabolic and mass transport models. Marcus et al. (2011) modeled the pH-profiles in dense mixed community biofilms and the effect of buffer type and capacity. This approach could be adapted for porous electrodes with modifications that consider the spatial variations of the relative diffusion coefficient and biofilm density that can be determined by magnetic resonance imaging (Renslow et al., 2010).

## 5. Conclusion

The present study reveals that the current production of MR-1 with the electrospun anode material ES300 is limited by proton transport to the bulk medium. Through modifications of the growth medium with higher buffer capacity the current density of 327 μA cm^-2^ could be increased 1.8-fold to a value of 590 μA cm^-2^. Riboflavin addition has limited effect on the current production of the electrospun material but enhances the current density of C-Tex 13 by stimulation of the biofilm attachment. The fact that we find the same (57.1 ± 5.2) μA mg^−1^ current to dry weight ratio with and without riboflavin addition shows that MR-1 forms biofilms to an extent that still allows for high metabolic activity. The current limit is set by the anode material (Erben et al., 2021b) and the chemical (micro)-environment (this work), mainly the local acidification. Utilizing growth media with high buffer concentrations and supplemented riboflavin in a production environment is unfavorable as costs are increased. The use of electrospun anode materials in a flow-through configuration may be a solution that enables high current densities while minimizing media costs.

## Data Availability Statement

The original contributions presented in the study are included in the article/Supplementary Material, further inquiries can be directed to the corresponding author/s.

## Author Contributions

JE carried out the experiments and wrote the manuscript with support from SK. ZP and ML carried out the preliminary tests. SK supervised the project. All authors contributed to the article and approved the submitted version.

## Conflict of Interest

ZP was employed by the company Electrochaea GmbH, Semmelweisstrasse 3, 82152 Planegg, Germany. The remaining authors declare that the research was conducted in the absence of any commercial or financial relationships that could be construed as a potential conflict of interest.
