# Supplementary material for: Local Acidification Limits the Current Production and Biofilm Formation of Shewanella oneidensis MR-1 With Electrospun Anodes
Source: Front Microbiol. 2021 Jun 14;12:660474. doi: 10.3389/fmicb.2021.660474 (PMC8236948; doi:10.3389/fmicb.2021.660474)
Supplement: Supplementary file 1 [file Data_Sheet_1.PDF]

Supplementary Material

1 BIOELECTROCHEMICAL REACTOR

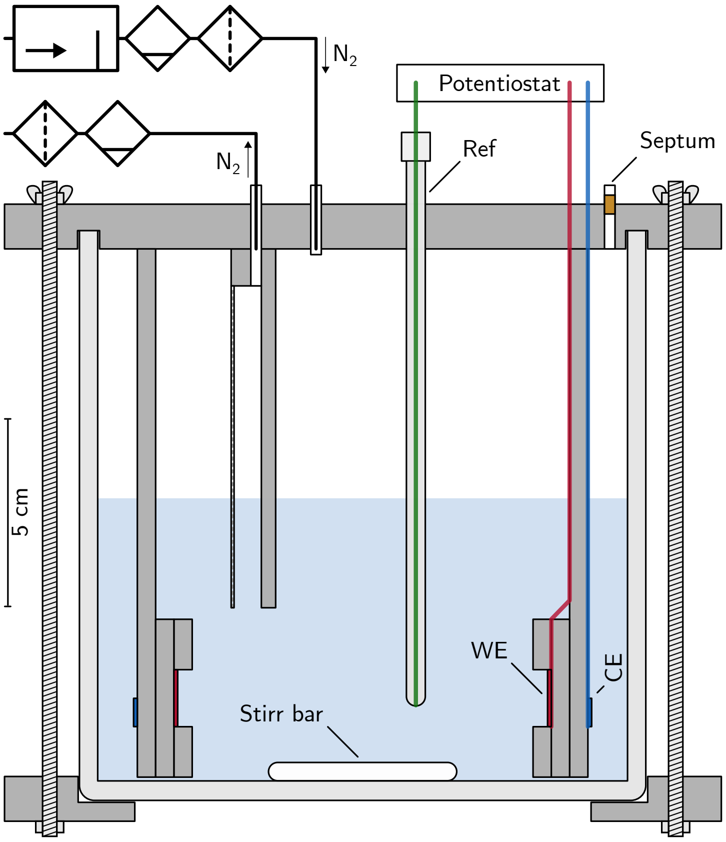

Figure S1. Schematic of the bioelectrochemical reactor with electrical peripherals and gas supply system. The working electrode is connected to the potentiostat in a two-wire configuration in order to reduce the uncompensated resistance. Abbreviations: working electrode (WE), counter electrode (CE), reference electrode (Ref).

2 (GROWTH) MEDIA

Table S1. Components of the different media. The symbol '–' indicates no change compared to the reference medium RM.

	Medium type					
Component	RM	25xPBS	100xRF	IM	AM	WB
Na_2HPO_4	10 mM	250 mM	–	40 mM	–	–
KH_2PO_4	1.76 mM	44 mM	–	7.04 mM	–	–
Sodium D/L lactate	50 mM	–	–	–	–	none
NaCl	137 mM	–	–	77 mM	–	–
KCl	2.7 mM	–	–	–	–	–
Casamino acids	$1\,g\,L^{-1}$	–	–	–	–	–
Riboflavin	none	–	100 µM	1 µM	–	–
Fumarate	none	–	–	–	100 mM	–
Trace elements	See Tab. S2	–	–	–	–	–

Table S2. Concentration of the trace elements in the media.

Component	Concentration
$(NH_4)_2SO_4$	1 mM
$CaCl_2$	0.1 mM
Mg_2SO_4	1 mM
$CoCl_2l$	5 µM
$CuSO_4$	0.2 µM
K_3BO_3	57 µM
$FeCl_2$	5.4 µM
$MnSO_4$	1.3 µM
Na_2EDTA	67.2 µM
Na_2MoO_4	3.9 µM
Na_2SeO_4	1.5 µM
$NiCl_2$	5 µM
$ZnSO_4$	1 µM

Table S3. Lysis buffer (LyB) composition. [a]Protease inhibitor cocktail for bacteria (Carl Roth, Germany)

Component	Concentration
Tris/HCl	20 mM
Triton X-100	16.5 mM
NaCl	137 mM
Protease inhibitors[a]	$25\,\mu L\,mL^{-1}$

3 CHRONOAMPEROMETRY DATA

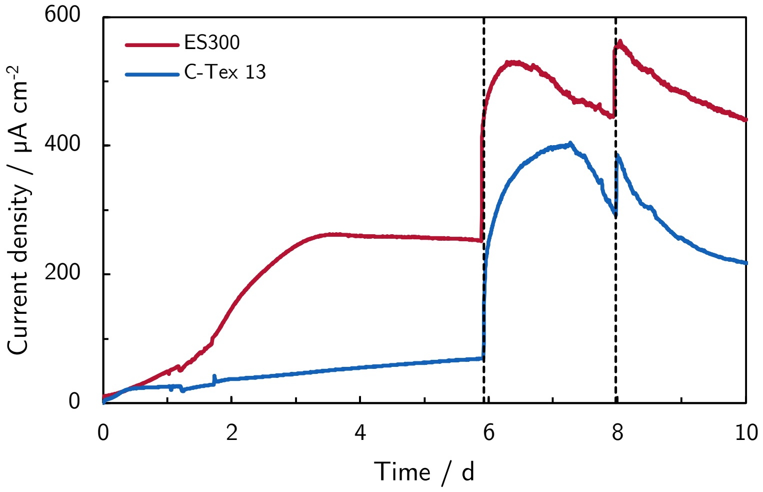

Figure S2. Current production with riboflavin spikes on day 6 (+500 nM) and 8 (+500 nM). The current does not stabilize within 2 days. Therefore, the effect of riboflavin on the current production is studied in individual experiments.

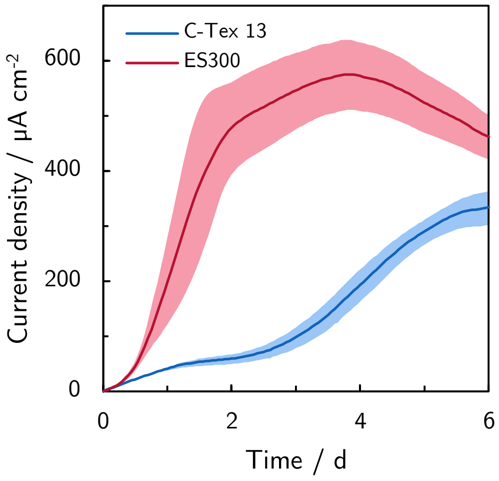

Figure S3. Current production with the improved medium (1 µM riboflavin and 40 mM PBS buffer). The shaded area corresponds to the sample standard deviation of three anodes.

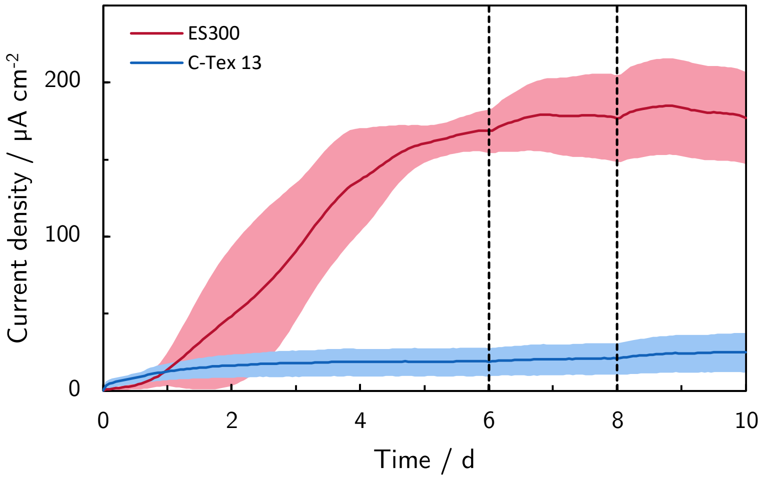

Figure S4. Current production with lactate spikes on day 6 (+25 mM) and 8 (+50 mM). The current density is evaluated before each lactate spike on day 6 and 8, and at the end of the experiment on day 10. The shaded area corresponds to the sample standard deviation of three anodes.

*

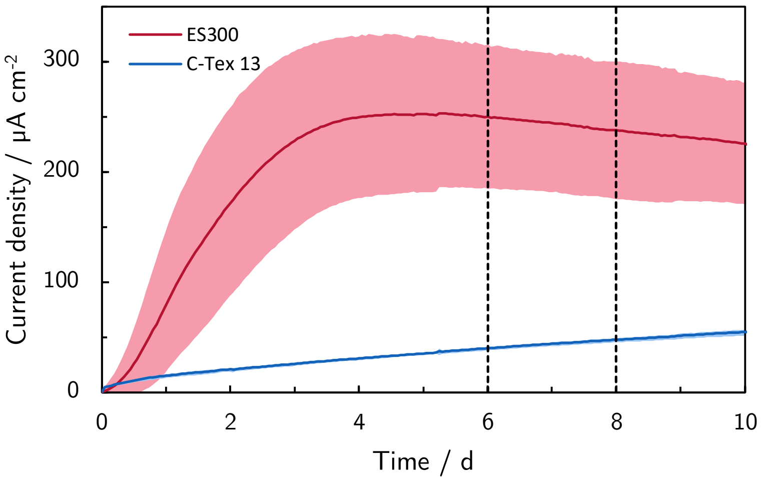

Figure S5. Control experiment for the spike experiments without pertubations. The current density is evaluated at on day 6 and 8, and at the end of the experiment on day 10. The shaded area corresponds to the sample standard deviation of four anodes.

Table S4. Experimental results as numerical values with sample standard deviation. Initial and final OD_{600} values are given in brackets.

**Riboflavin**		0 nM (0.05 / 0.05)	500 nM (0.04 / 0.04)	1000 nM (0.05 / 0.04)
ES300	i_{Max} / µA cm^{-2}	217.5 ± 9.8	254 ± 35	326 ± 41
	i_{Final} / µA cm^{-2}	199 ± 13	200 ± 22	248 ± 20
	Dry weight / mg	5.51 ± 0.99	4.76 ± 0.45	5.42 ± 0.67
	Fold change (i_{Max})	–	1.17 ± 0.16	1.50 ± 0.20
	Fold change (i_{Final})	–	1.00 ± 0.11	1.25 ± 0.13
C-Tex 13	i_{Max} / µ A cm^{-2}	60.0 ± 3.2	207 ± 10	353 ± 19
	i_{Final} / µ A cm^{-2}	59.2 ± 3.1	204.8 ± 9.8	334 ± 15
	Dry weight / mg	2.089 ± 0.073	7.23 ± 0.95	13.9 ± 2.8
	Fold change (i_{Max})	–	3.45 ± 0.17	5.89 ± 0.32
	Fold change (i_{Final})	–	3.46 ± 0.17	5.64 ± 0.26
**Buffer capacitance** (0.07 / 0.05)		10 mM	+10 mM	+20 mM
ES300	Current density / µA cm^{-2}	327 ± 37	412 ± 56	590 ± 25
	Dry weight / mg	–	–	12.1 ± 1.8
	Fold change	–	1.260 ± 0.041	1.8 ± 0.2
C-Tex 13	Current density / µA cm^{-2}	83 ± 31	88 ± 34	88 ± 37
	Dry weight / mg	–	–	5.1 ± 1.9
	Fold change	–	1.051 ± 0.023	1.213 ± 0.019
**Inoculation cell density** (0.06 / 0.20)		0.05	+0.05	+0.10
ES300	Current density / µA cm^{-2}	176 ± 22	188 ± 15	188 ± 25
	Dry weight / mg	–	–	5.7 ± 2.0
	Fold change	–	1.068 ± 0.038	1.214 ± 0.090
C-Tex 13	Current density / µA cm^{-2}	19.90 ± 0.86	24.2 ± 3.7	24.21 ± 0.84
	Dry weight / mg	–	–	1.823 ± 0.065
	Fold change	–	1.223 ± 0.038	2.088 ± 0.090
**Lactate** (0.05 / 0.04)		25 mM	+25 mM	+50 mM
ES300	Current density / µA cm^{-2}	167 ± 14	177 ± 28	178 ± 29
	Dry weight / mg	–	–	4.6 ± 1.2
	Fold change	–	1.046 ± 0.097	1.05 ± 0.11
C-Tex 13	Current density / µA cm^{-2}	19.0 ± 9.2	21 ± 10	25 ± 13
	Dry weight / mg	–	–	1.491 ± 0.098
	Fold change	–	1.1089 ± 0.0060	1.306 ± 0.049
**Control** (0.05 / 0.05)		Day 6	Day 8	Day 10
ES300	Current density / µA cm^{-2}	250 ± 66	237 ± 63	226 ± 55
	Dry weight / mg	–	–	7.2 ± 2.3
	Fold change	–	0.950 ± 0.025	0.906 ± 0.030
C-Tex 13	Current density / µA cm^{-2}	40.0 ± 1.6	47.3 ± 2.3	53.9 ± 9.4
	Dry weight / mg	–	–	3.2 ± 1.1
	Fold change	–	1.185 ± 0.048	1.350 ± 0.062
**Improved medium** (0.06 / 0.06)				
ES300	i_{Max} / µA cm^{-2}	575 ± 63		
	i_{Final} / µA cm^{-2}	461 ± 40		
	Dry weight / mg	9.50 ± 0.60		
C-Tex 13	i_{Max} / µA cm^{-2}	334 ± 32		
	i_{Final} / µA cm^{-2}	334 ± 32		
	Dry weight / mg	7.97 ± 0.68		
